# Characterization, antioxidant and immunomodulatory potential on exopolysaccharide produced by wild type and mutant *Weissella confusa* strains

**DOI:** 10.1016/j.btre.2018.e00271

**Published:** 2018-07-03

**Authors:** Bukola Adebayo-Tayo, Racheal Ishola, Titiloye Oyewunmi

**Affiliations:** a Department of Microbiology, University of Ibadan, Ibadan, Oyo State, Nigeria; b Immunology Unit, Department of Chemical Pathology, College of Medicine, University of Ibadan, Nigeria

**Keywords:** Exopolysaccharide, Immunomodulatory, Antioxidant, Immunoglobulin, Lactic acid bacteria

## Abstract

Characterization, antioxidant and immunomodulatory potential of exopolysaccharide (EPS) produced by wild type and mutant *Weissella confusa* was investigated. The EPS production ranged from 5490.2 to 5580.7 mg/L. Wild type *Weissella confusa* (WWCEPS) had the highest EPS production. Eight (8) sugar moieties were present in the EPS. Galactose had the highest EPS composition (34.6 mg/100 g and 33.5 mg/100 g EPS) in Wild type *Weissella confusa* EPS (WWCEPS) and mutant *Weissella confusa* EPS (MWCEPS). Wild type *Weissella confusa* and mutant *Weissella confusa* EPS had antioxidant capacity. The scavenging assay for the antioxidant increased in a dose dependent (0.5–10 mg/mL) manner. Wild type *Weissella confusa* EPS had the highest 1,1-Diphemy 1-2-picryl-hydrazyl (DPPH) capacity, total antioxidant activity, hydrogen peroxide and reducing power activity (71%, 1.9%, 86.7% and 1.9%). The mice injected peritorially with mutant *Weissella confusa* EPS had the highest IgG and IgM (68–87 mg/dL and 64–70 mg/dL). IgA of the mice treated with Wild type *Weissella confusa* EPS increased from 67 to 73 mg/dL. Wild type and mutant *Weissella confusa* EPS had immunomodulatory activity on the treated mice.

## Highlights

• Characterization, antioxidant and immunomodulatory potential of exopolysaccharide (EPS) produced by wild and mutant *Weissella confuse* was evaluated.
• Wild *Weissella confusa* (WWCEPS) produced the highest EPS compare to the mutant strain.
• The EPS were heteropolysaccharides containing eight (8) monosaccharides in which galactose had the highest composition (34.60 mg/g and 33.47 mg/g EPS) in wild *Weissella confusa* EPS (WWCEPS) and mutant *Weissella confusa* EPS (MWCEPS).
• WWCEPS and MWCEPS had antioxidant capacity and WWCEPS had the highest DPPH capacity, total antioxidant activity, hydrogen peroxide and reducing power activity (71%, 1.88%, 86.7% and 1.85%).
• Wild and mutant *Weissella confusa* EPS exhibited immunomodulatory activity on the treated mice.

## 1 Introduction

Microbial exopolysaccharides (EPS) comprise a wide group of polymers secreted by most microorganisms that are either tightly attached to the cell surface (capsular polysaccharides) or released as extracellular slime in the surroundings of the cell [1]. Several studies have been performed on exopolysaccharides (EPS) produced by lactic acid bacteria (LAB) during the last decade [[2], [3], [4], [5], [6]]. Exopolysaccharides exhibit a wide spectrum of applications in pharmaceutical, biomedical and food industries. In food industries, EPS are widely used as thickeners, stabilizers, emulsifier and gelling agents. They have been shown to improve the rheology and texture of fermented foods. EPS have the ability to improve human health due to its antioxidant, antitumor, antiulcer, immunomodulating or cholesterol-lowering activities [7,8]. EPS also help probiotics to survive the gastric acid and bile salts in the gastrointestinal tract [9]. The increased viscosity of foods containing EPS contribute to an increase in the residence time of ingested fermented milk in the gastrointestinal tract, thereby supporting transient colonization by probiotic bacteria [10].

Reactive Oxygen Species (ROS) such as superoxide anion (O_2_^−^), hydrogen peroxide (H_2_O_2_) and hydroxyl radical are produced during cellular metabolism as a result of oxidative stress. Oxidative stress is an imbalance that occurs between the systematic manifestations of Reactive Oxygen Species (ROS) and the ability of a biological system to repair the resulting damage or readily detoxify the reactive intermediates [11]. ROS are produced from molecular oxygen of normal cellular metabolism. This leads to disruptions in normal mechanisms of cellular signaling, base damage as well as strands breakage in DNA. These ROS play important roles in cell signaling, gene expression, apoptosis and ion transportation [12]. When the ROS are deficient or generated in excess, biomolecules including nucleic acids, lipids and protein can be damaged by the oxidative stress process.

The significant pathological role of ROS in human diseases such as cancer, cirrhosis, Alzheimar’s disease, Parkinson’s disease, atherosclerosis, and arthritis has been reported [13,14]. Antioxidants are substances which can delay or inhibit oxidation. Antioxidants function in several ways including preventing the formation of radicals, scavenging free radicals, formation of hydrogen peroxide and other peroxides [15]. Oxygen derived radical are highly reactive and are formed by exogenous chemicals or endogenous metabolic processes in the human body [11]. They can also be formed in food systems and they are capable of oxidizing biomolecules resulting in tissue damage and cell death. Enzymes such as superoxide dismutase or compounds such as glutathione, ascorbic acid and tocopherol protect almost all organisms against free radical damage leave out by enzymes [16]. Some synthetic antioxidants such as butylated hydroxytoluene (BHT), *n*-propyl gallate (PG) and butylated hydroxyanisole (BHA) have strong antioxidant activity against several oxidation systems, thereby posing potential risks in human system such as liver damage and cancer [17].

Exploitation of safer and natural antioxidants from bio-resources such as exopolysaccharide that can replace synthetic antioxidants has received a great deal of attention in recent years [18]. EPS from LAB usually have low cytotoxicity and side effects, which make them good candidates for immunotherapy against cancer and as anti-oxidants [19,20].

The ability of LAB strains to enhance cell-mediated immune responses, including T-lymphocyte proliferation, mononuclear cell phagocytic capacity, and natural killer (NK) cell tumoricidal activity has been reported [21]. It can also enhance humoral immunity which is mediated by immunoglobulins (IgA, IgG, IgM, IgE and IgD) produced by bone marrow-derived lymphocyte (B lymphocytes) and it is responsible for specific recognition and elimination of extracellular antigens.

Mutagenesis has been reported as a classical method of strain improvement widely employed in food industry to improve microorganisms with desirable qualities [22,23]. Through mutagenesis the genome may be modified to increase the potential yield of a desirable product.

Mutant *Weisella confusa* is a strain with improved characteristics obtained by exposure of the wild type *Weisella confusa* to UV radiation. The genetic composition of the mutant strains has been modified as a result of random mutagenesis. It has higher exoploysaccharides producing ability than the wild type [24].

Ability of *Weissella* species to produce EPS that are useful in food industries as well as other applications has been reported [25,26]. EPS from LAB enhances host immunomodulatory functions via activation of macrophages and lymphocytes [27]. These immunomodulatory activities have been suggested to be mediated via interactions between immune cells and intact bacterial cells or LAB components such as peptidoglycan, teichoic acid, and exopolysaccharide. The objective of this study is to characterized EPS produced by wild type and mutant *W. confusa,* determine the antioxidant and immunomodulatory potential of the EPS.

## 2 Materials and methods

### 2.1 Culture collection

The wild type and mutant *Weissella confusa* used in this experiment were collected from the culture collection of our previous work in Microbial Physiology Unit, Department of Microbiology, University of Ibadan [24]. The isolates was maintained in De Man, Rogosa and Sharpe (MRS) broth [28] and stored at 28 °C for three days.

### 2.2 Production of exopolysaccharides by the LAB strains

Seed culture of wild type and mutant *Weissella confusa* were prepared by transferring 0.5 mL of the stock frozen culture to 10 mL of MRS broth and incubated for 16 h at 30 °C. The resulting culture was transferred (2% v/v) into Modified Exopolysaccharide Selection Medium (mESM) [29]. The medium contains 5% skimmed milk (Oxoid), 0.35% yeast extract (Oxoid), 0.35% peptone (Difco) and 5% glucose (BDH) and incubated at 30 °C for 16 h.

#### 2.2.1 Extraction and quantification EPS

The fermentation medium was centrifuged at 15,000 × *g* for 15 min. at 4 °C. The EPS was precipitated at 4 °C by the addition of 2 vol. of ethanol (100%). The resulting precipitate was collected after centrifugation (15,000 × *g* for 15 min. at 4 °C).

#### 2.2.2 Determination of total sugar of the EPS samples

The total sugar concentration was determined by phenol-sulfuric acid method using glucose as a standard [30]. 0.1 mL of EPS samples was diluted in 2.0 mL of distilled water. 1.0 mL of 6% phenol and 5.0 mL of sulfuric acid 95% (v/v) was added to the solution and shaken up after 10 min standing, and then absorbance at 490 nm was measured. The concentration of EPS was determined in triplicate and 2.0 mL distilled water used as blank. The EPS content of each sample was calculated by the standard curve. The glucose standard curve was prepared for the quantitative determination, according Dubois et al. [31] with some modifications.

#### 2.2.3 FTIR analysis of the EPS samples

The EPSWWC and EPSMWC were analyzed using FTIR [32]. 2 mg of the EPS were ground with 200 mg dry KBr. The pressing were pressed into a 16 mm diameter mold. The Fourier transform-infrared (FT-IR) spectra were recorded on a Shimadzu IR Affinity 1S instrument with a resolution of 4 cm^−1^ in the 4000–400 cm^−1^ region.

### 2.3 Determination of the monosaccharide composition of the EPS produced by wild type and mutant *Weissella confusa* using High Performance Liquid Chromatography

Monosaccharides composition of the EPS was determined using HPLC equipped with Refractive Index detector. The EPS samples was dissolved in water, heated to rehydrate and hydrolysed using 2 N trifluroacetic acid (TFA) at 120 °C for 1 h in sealed glass tubes. After hydrolysis the tubes were opened, evaporated to dryness, methanol was added to remove TFA [33]. The monosaccharide composition of the hydrolysed samples was determined using Beckman UI- transphere ODSC -18 (250 nm × 4.6 mm i.d.), 18% (v/v) acetonitrile in 120 mM aqueous acetic acid as mobile phase at 1 mL/min flow rate using refractive index detector. The amount of sugars was determined by measuring the areas under the curves with an integrator and by comparing various sugar standards of a known retention times to that of the samples [34].

### 2.4 Determination of anti-oxidant potential of the EPS produced by wild type and mutant *Weissella confusa*

The 1,1-Dipheny 1-2-picryl-hydrazine (DPPH) scavenging potential of the EPS samples was done using Blois [35] method as modified by Gulcin [36]. EPS at different concentrations (50, 100, 250, 500 and 1000 mg/mL) was mixed with 3 mL of ethanol and mL of 0.1 mM DPPH solution was added. The reaction mixture was kept in the dark for 30 min. Absorbance was taken at 517 nm and ascorbic acid was used as standard. Reduction in absorbance indicates DPPH radical scavenging activity [37].

The reducing power of the EPS was evaluated using the method of Oyaizu [38] with slight modification by Gulcin et al. [39]. Different concentration of EPS (200–1000 mg/mL) in 1 mL of distilled water was added to 2.5 mL of 2 mM phosphate buffer (pH6.6) and 1% of Potassium ferricyanide (2.5 mL). The mixture was kept at 50 °C for 20 min. 10% trichloroacetic acid (2.5 mL) was added to the reaction mixture and spin at 3000 rpm for 10 min. Distilled water (2.5 mL) and 1% ferric chloride (0.1 mL) was added. The Absorbance was taken at 700 nm after 10 min of incubation and ascorbic acid was used as standard.

The Hydrogen peroxide scavenging potential of the EPS was done using standard method [40]. 10 mM solution of Hydrogen peroxide was prepared in 0.1 M phosphate buffered saline (pH 7.4). Different concentration (50, 100, 250, 500 and 1000 mg/mL) of EPS samples prepared. From each concentration, 1 mL of the prepared EPS samples was added to 2 mL of the hydrogen peroxide solution. After 10 min of incubation at 30 °C, Absorbance of the reaction mixture was taken at 230 nm after 10 min against blank solution using UV–vis spectrophotometer. Ascorbic acid was used as standard.

Total antioxidant activity of EPS was determined using method of Mitsuda et al. [41] as reported by Kanamarlapuda and Muddada [42]. Mixture of 1.235 g of ammonium molybtate (4 mM), 0.6 M Sulfuric acid (45 mL), 0.9942 g of Sodium sulphate (28 mM) and 250 mL with distilled water was used as total antioxidant capacity. 0.1 mL of EPS from different concentration (50, 100, 250, 500 and 1000 mg/mL) was mixed in 1 mL total antioxidant capacity and absorbance was taken at 695 nm after 15 min incubation. Ascorbic acid was used as standard.

### 2.5 Determination of immunomodulatory potential of the EPS produced by wild type and mutant *Weissella confusa*

#### 2.5.1 Animals and rearing conditions

Thirty female Swiss albino mice (8–10 weeks of age, weighing 20–24 g) were maintained in the Animal Breeding Unit, University College Hospital, Ibadan. All mice were fed with rat pellets and given water ad libitum and allowed to acclimatize to the laboratory environment for two weeks prior to the experiment. All the procedures used in this study conformed to the guidelines for care and use of animals in research and teaching.

#### 2.5.2 Grouping of the experimental animal

The mice were divided into the following five groups (n = 5) as follows: Group 1 – blank control, Group 2 – SRBC control, Group 3 – EPS produced by wild type strain *W. confusa* (EPSWCWS), Group 4 – EPS produced by mutant strain *W. confusa* (EPSWCMS).

#### 2.5.3 Effect of the exopolysaccharides on experimental animal

Investigation of the effect of EPS on the experimental animal was done using the modified method Majolagbe et al. [43]. Group 3 and 4 of six weeks old female mice were injected intraperitonially (IP) with 10 mg of the EPS. On the same day, mice were immunized with 0.1 mL of Sheep Red Blood Cell (SRBC) diluted to 10% concentration with 0.85% sterile NaCl as antigen. After 24 h, these mice were given a second IP injection of 10 mg of the EPS sample. Two groups (Group 1 and 2) of control animals were also included. One of the groups was given only 0.1 mL IP injection of SRBC as antigen while the other group receives 0.1 mL DMSO. On 4th–5th day of the SRBC immunization, the mice were sacrificed by cervical dislocation and the spleens were removed. With the help of wire gauze, the spleens were processed into single cell suspension mesh in Petri dish. The cell suspension in Eagle’s medium was then centrifuged at 2000 rpm for 5 min. The pellet was washed twice and finally suspended in 1 mL Eagle’s medium. Two tenfold dilution was made and the cells were counted in a hemocytometer and cell viability was determined using trypan exclusion. The cells were kept in ice until used.

#### 2.5.4 Determination of immunoglobulin

The assay for antibodies was based on turbidimetric measurement. Turbidity was caused by the formation of antigen-antibody insoluble immune complexes. For the determination of IgG, IgA and IgM, the following reagents were used: Blood serum, (phosphate buffer saline-pH 7.4), IgG,

IgA and IgM Antibody, Sodium azide and distilled water. The IgG, IgA and IGM of the treated and untreated mice were determined by diluting the blood serum samples and the control in 0.9% saline (1:10). 20 μL of the diluted samples was added to 900 μL of phosphate buffer and labeled sample A2. The absorbance of sample A1 was taken at 340 μL. 100 μL antibody reagents was added into the prepared samples and mixed properly. The reaction mixture was incubated for 15 min. Absorbance of A2 and control was taken at 340 nm.

### 2.6 Statistical analysis

All experiments were performed in triplicates and the results were statistically subjected to Analysis of Variance (ANOVA) using SPSS (version 11.0, Chicago, IL). Probability values (P = 0.05) was considered significant to indicate difference. Duncan’s Multiple Range Test (P = 0.05) were used to determined the significant difference.

## 3 Results and discussion

### 3.1 Production of EPS by the wild type and mutant *W. confusa*

The EPS produced by the LAB strain is shown in Table 1. The EPS production by the wild type and mutant *Weissella confusa* ranged from 5490.16 to 5580.72 mg/L. WWC produced the highest EPS. EPS production by *W. confusa* is in line with the report of De Vuyst and Degeest [9].

**Table 1:** Production of EPS by the wild type and mutant *Weissella confuse*.

LAB Isolates	Isolate Code	EPS production (mg/L)
Wild type *Weissella confusa*	EPSWWC	5490.2
Mutant *Weissella confusa*	EPSMWC	5580.7

Glucose and glucose moiety was reported as source of sugar for biosynthesis of heteropolysaccharides [44]. Mozzi et al. [45] reported the production of heteropolysaccharides by lactic acid bacteria.

**Fig. 1:**
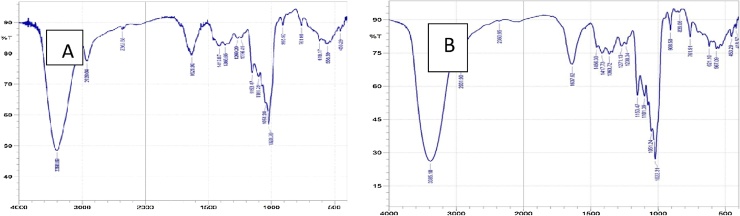
FTIR spectra of EPSWWC and EPSMWC.

The FTIR spectra of EPSWWC and EPSMWC are shown in Fig. 1a–b. The distribution of functional groups present in EPSWWC and EPSMWC is shown in the Table 2.

**Table 2:** Distribution of functional group of the FTIR spectra of EPSWWC and EPSMWC.

S/N	Peak (cm^−1^)	Functional Group	EPSWWC	EPSMWC
1	418.57	Chloroalkanes	−	+
2	453.29	Chloroalkanes	−	+
3	553.59; 567.09	Alkylhalide	+	+
4	619.17; 621.10	Alkynes	+	+
5	761.91	Alkanes	+	+
6	839.06	Alkanes	−	+
7	908.5	Alkenes	+	+
8	1020.38;1022.31	Alkenes	+	+
9	1051.24	Alkenes	+	+
10	1101.39	Alkanes	−	+
11	1153.47	Alkenes	+	+
12	1236.41; 1238.34	Secondary amines	+	+
13	1269.2; 1271.13	Alkyl halide	+	+
14	1365.65; 1363.72	Alkanes	+	+
15	1413.87; 1417.73	Esters	+	+
16	1456.30	Aromatic	−	+
17	1629.9; 1637.62	Aldehyde	+	+
18	2362.88; 2360.95	Aldehyde	+	+
19	2828.04; 2931.90	Aldehyde	+	+
20	3385.18; 3398.69	Hydroxyl/alcohol	+	+

Similarities in the absorption peaks around 3000 cm^1^ characteristics of carbon- and hydrogen- containing species was observed in the EPSWWC and EPSMWC as shown in Fig. 1a–b. Absorptions in the region of 3385.18 cm^1^ and 3398.69 cm^−1^ indicate that the compound is likely to be unsaturated or aromatic while peaks at 1629.9 cm^1^ and 1641.48 cm^−1^ further highlight the presence of un-saturation in the EPS. The EPSWWC differ from EPSMWC in four peaks less in the regions 453.29–908.50 cm^1^, 1236.41–1641.48 cm^1^ and 2061.97–3398.09 cm^1^. This indicates lesser OH presence in the EPSWWC as this region is characteristics of alcohol and aromatic bonds [46].

### 3.2 Monosaccharide composition of the EPS

**Fig. 2:**
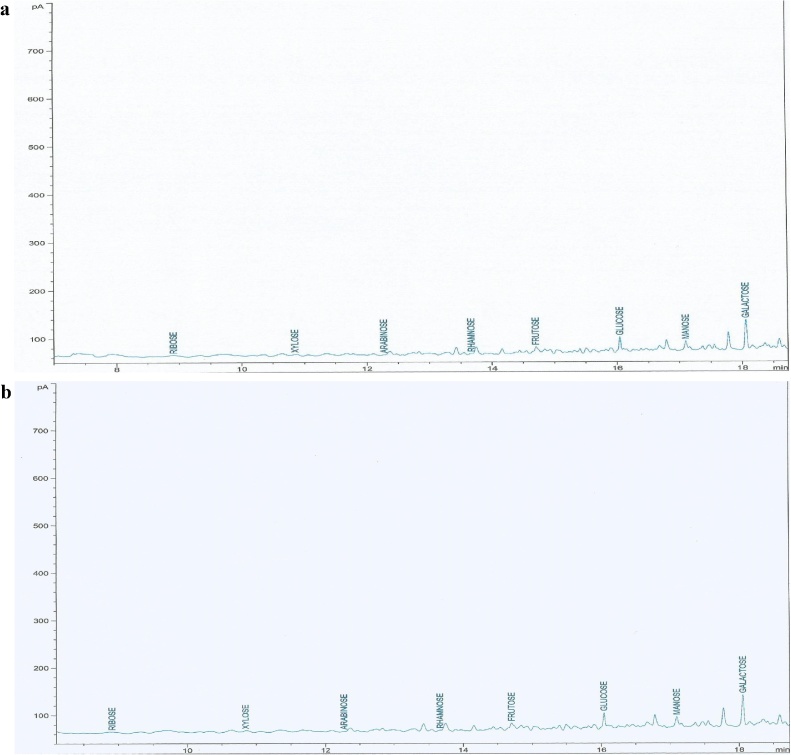
a–b Chromatogram of the EPS produced by (a) wild *W. confusa* (EPSWWC) and (b) mutant *W. confusa* (EPSMWC).

The chromatogram of the monosaccharide composition of the EPS samples is shown in Fig. 2a and b. The monosaccharide composition and sugar content of the EPS produced by wild type and mutant *W. confusa* is shown in Table 3a, Table 3b. It was observed that all the EPS samples produced by both wild type and mutant strain of *W. confusa* had galatose, mannose, glucose, fructose, rhamnose, arabinose, xylose and ribose sugars. There was a significant difference (P ≤ 0.05) in sugar content of the EPS produced by wild and mutant *Weissella confusa* (WWC and MWC). The sugar concentration ranged from 1.09–34.62 mg/g and 2.07–33.47 mg/g. Galactose had the highest content followed by glucose while ribose had the least. However, glucose and galactose had the highest content in EPS produced by wild type and mutant *Weissella confusa.* Arabinose and ribose had the least.

**Table 3a:** Monosaccharide composition and sugar concentration of EPS produced by wild *W. confusa* (EPSWWC).

Monosaccharide name	Retention Time	Area under curve	Amount (mg/g)
Ribose	8.92	3.84	1.77
Xylose	10.65	4.43	3.15
Arabinose	12.27	4.44	1.09
Rhamnose	13.74	2.82	3.20
Fructose	14.70	6.96	8.53
Glucose	16.04	1.37	19.98
Mannose	17.09	7.16	1.32
Galactose	18.06	1.24	34.60

**Table 3b:** Monosaccharide composition and sugar concentration of EPS produced by mutant *W. confusa* (EPSMWC).

Monosaccharide name	Retention Time	Area under curve	Amount (mg/g)
Ribose	8.92	3.84	2.07
Xylose	10.65	4.43	3.24
Arabinose	12.27	4.44	7.26
Rhamnose	13.74	2.82	3.55
Fructose	14.70	6.96	9.59
Glucose	16.04	1.37	18.18
Mannose	17.09	7.16	1.79
Galactose	18.06	1.24	33.47

The EPS produced by WWC and MWC were heteropolymeric in nature. The present of 8 sugars in the EPS of wild type and mutant *Weissella confusa* was an indication that the EPS was heteropolysaccharide. Sugar standard of the same retention time was used to determine the monosaccharide’s composition of the samples [31].

### 3.3 Determination of the antioxidant potential of the EPS produced by the LAB

**Fig. 3:**
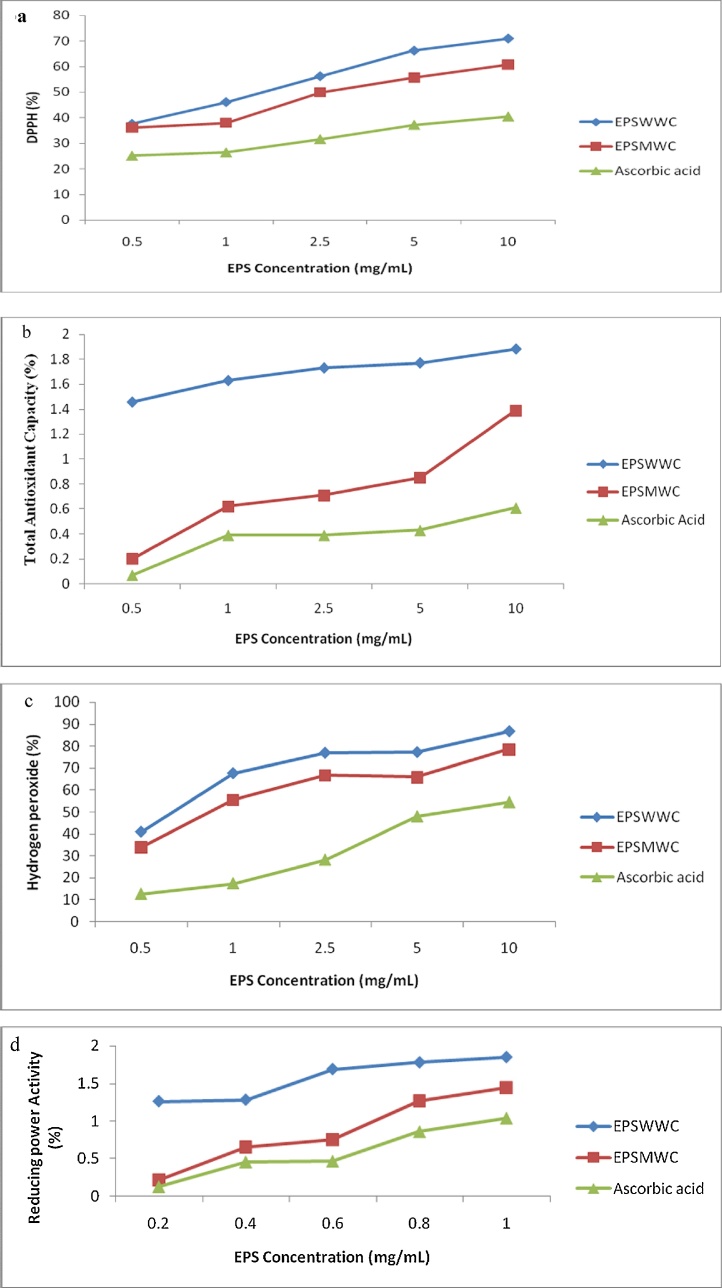
a. Picrylhydrazyl free radical (DPPH) radical scavenging assay (%) of the EPS produced by wild type *W. confusa* (EPS) and mutant *W. confusa* (EPS). b. Total antioxidant capacity (%) of the EPS produced by wild type *W. confusa* (EPS) and mutant *W. confusa* (EPS). c. Hydrogen peroxide Capacity (%) on EPS produced by wild type *W. confusa* (EPS) and mutant *W. confusa* (EPS). d. Reducing power concentration on EPS produced by wild type *W. confusa* (EPS) and mutant *W. confusa* (EPS). Key: EPSWWC- EPS produced by wild *Weissella confusa*, EPSMWC- EPS produced by mutant *Weissella confusa*.

The antioxidant capacity (OH—, DPPH, Total antioxidant capacity and reducing power capacity) of EPS produced by LAB using different EPS concentrations is shown in Fig. 3a–d.

#### 3.3.1 DPPH scavenging activity of the EPS produced by the LABs

The DPPH scavenging activity of the EPS produced by the wild type and mutant *W. confusa* is shown in Fig. 3a. The DPPH activity increased with increase in EPS concentration. At concentration of 0.5–1.0 mg/mL, the DPPH activity ranged from 37.71 to 71% and 36.1 to 60.7% for the EPSWWC and EPSMWC. From the DPPH activity, it was observed that the EPS samples exhibited a higher scavenging activity compared to ascorbic acid. The EPS from the WWC and MWC exhibited higher scavenging activity than ascorbic acid.

This showed that EPS produced by wild type and mutant *W. confusa* donates hydrogen ions to react with the DPPH radical. Ascorbic acid used as control had the least antioxidant activity. This could be as a result of the bioactivities of polysaccharides which could be influenced by molecular weight, chemical components, and extraction methods [47]. The EPS have been demonstrated to play an important role as free radical scavengers in the prevention of oxidative damage in living organisms and can be explored as novel potential antioxidant. DPPH is a stable free radical with a maximum absorbance at 517 nm in ethanol. When DPPH encounters a proton-donating substance such as an antioxidant, the radical is scavenged and the absorbance is reduced [48].

#### 3.3.2 Total antioxidant capacity scavenging assay of the EPS produced by the LABs

Total antioxidant activity of the EPS produced by the LABs is shown in Fig. 3b. At 0.5 and 1.0 mg/mL, the total antioxidant activity ranged from 1.46 to 1.8 % and 0.2 to 1.39%. EPSWWC had the highest total antioxidant capacity while the ascorbic acid used as control had the least antioxidant activity. This could be due to the fact that total antioxidant activity of a polysaccharide depends primarily on its structural characteristic and configuration of the glycosidic bond as reported by Zheng et al. [49] and is thus not a function of a single factor but a combination of several factors. It could also be because of some activities which may be due to the presence of other antioxidant components in the crude EPS such as proteins, peptides and microelements, thereby exhibiting potent antioxidant efficacy by interacting with other compounds present in the crude EPS.

#### 3.3.3 Hydrogen peroxide scavenging assay of the EPS produced by the LABs

Hydrogen peroxide activity of the EPS produced by the LABs is shown in Fig. 3c. At 0.5 and 1.0 mg/mL, H_2_O_2_ scavenging activity ranged from 40.9 to 86.7% and 33.7 to 78.6% for EPSWWC and EPSMWC. The highest capacity was recorded in ESPWLD while the least was exhibited in EPSMWC. At 2.5, 5 and 10 mg/mL, the activity ranged from 64.2 to 80.7%, 65.7 to 81.0% and 78.6 to 88.5%. The highest capacity was exhibited in EPSWLD and the least was observed in EPSMWC. Ascorbic acid used as control had the least antioxidant activity.

Sahu and Gray [50] reported that free ferrous iron is quite sensitive to oxygen which results in superoxide and ferric iron. The reaction generates into hydrogen peroxide. Reaction of ferrous ion with superoxide hydrogen peroxide results in fenton reaction which leads into a hydroxyl radical by oxidizing biomolecules in the surroundings. The hydroxyl radical production is directly related to the concentration of copper or iron [50].

#### 3.3.4 Reducing power capacity scavenging assay of the EPS produced by the LABs

The reducing power activity of the EPS produced by EPSWWC and EPSMWC is shown in Fig. 3d. At 0.2–1.0 mg/mL, the reducing power activity ranged from 0.26 to 1.85% and 0.21 to 1.44% respectively. ESPWWC had the highest activity. Generally, the highest antioxidant scavenging activity was recorded at 10 mg/mL EPS concentration for all the EPS produced by the EPSWWC and EPSMWC strains. All concentrations of the EPS showed higher activities than ascorbic acid which is the control. The ascorbic acid used as control had the least antioxidant activity. This result shoes that EPS ferric ions wad labeled with reducing ability along with the properties of an electron donor for neutralizing free radicals by forming a more stable product. According to the findings of Liu and Pan [51], the EPS synthesized by *L. paracasei* and *L. plantarum* have a reductive activities using K_3_Fe (CN)_6_ reduction method.

The EPS produced by wild type *W. confusa* (EPSWWC) exhibited higher antioxidant capacity than EPS produced by mutant *W. confusa* (EPSMWC). The variation in antioxidant capacity of the EPSWWC and EPSMWC may be due to the genetic modifications that occurred in the LAB as a result of random mutagenesis [23].

### 3.4 Determination of the immunomodulatory potential of the EPS produced by the LABs

The immunomodulatory potential of the EPS produced by the LABs on mice is shown in Table 4. There was a significant different (P = 0.05) in the immunoglobulin level of the experimental mice. The immunoglobulin level was detected using Sheep Red Blood Cell (SRBC). IgA level ranged from 67 ± 0.03 to 73 ± 0.24 mg/dL. Group 2 had the highest IgA level followed in order by group 3 (mice treated with EPS from wild type *Weissella confusa*) and Group 4 (mice treated with EPS from mutant *Weissella confusa*). The least IgA was recorded in group 1 (control).

**Table 4:** Immunomodulatory activity of EPS produced by the LAB strains.

Groups	Treatments			Immunoglobulin (mg/dL)		
	SRBC	WEPS	MEPS	IgG	IgA	IgM
Grp 1 (Blank)	−	−	−	68 ± 0.03^f^	67 ± 0.03^e^	64 ± 0.03^d^
Grp 2(c1)	+	−	−	94 ± 0.03^a^	91 ± 0.24^a^	71 ± 0.11^b^
Grp 3	−	+	−	75 ± 0.08^b^	85 ± 0.14^b^	63 ± 0.41^e^
Grp 4	−	−	+	87 ± 0.24^c^	73 ± 0.21^c^	70 ± 0.21^c^

Each value is a mean ± standard error of three replicates. Values in the same column with different letters as superscripts are significantly different by Duncan multiple range test (P < 0.05).

**Key:** Group 1 – Control. Grp 2 – Mice given SRBC. Grp 3 – Mice given Exopolysaccharide produced by wild type strain of *Weissella confusa* (EPSWWC). Grp 4 – Mice given Exopolysaccharide produced by mutant strain of *Weissella confusa* (EPSMWC).

The IgG level of the mice ranged from 68 ± 0.03 to 94 ± 0.03 mg/dL. Group 2 (mice with SRBC) had the highest which was followed in order by the IgG of Group 4 (mice treated EPS from with mutant *Weissella confusa*) and Group 4 (mice treated EPS from with wild type *Weissella confusa*). The least IgA was recorded in group 1 (control).

The IgM level of mice ranged from 63 ± 0.09 to 70 ± 0.05 mg/dL. Group 2 (mice with SRBC) have the highest IgM level followed in order by the IgM of Group 4 (mice treated EPS from with mutant *Weissella confusa*) and group 3 (mice treated with EPS from wild type *Weissella confusa*) had the least IgM.

However, IgA had the highest immunoglobulin concentration while the least was IgM. Treatment of mice with EPS from wild type *Weissella confusa* (Group 3) favoured the highest production of immunoglobulin when compare to group 2 (mice with SRBC). The immunoglobulin level of mice is higher in all other groups compare to mice in group 1 (control).

Ability of EPS produced by wild type and mutant *Weissella confusa* to stimulate IgG in the immune system of the mice may be due to the engulfment of macrophages by the EPS. There is a stimulation of serum glycoproteins to produce a subpopulation of white blood cells called lymphocytes., Zhu et al. [52] and Qiu et al. [53] reported that several studies have demonstrated the effect of LAB intake on improved host immunity via the production of IgA. Yang et al. [54] also reported enhanced serum levels of IgA, IgM and IgG in broiler chickens after being fed the LAB for 40 days. More promisingly, serum levels of IgA, IgG and IgM of colorectal cancer patients who had undergone elective laparoscopic radical surgery, significantly increased after Introduction of LAB. Qiu et al. [53] reported that one-day old broilers fed a diet containing *Lactobacillus casei*, *Bifidobacterium bifidium*, and *Enterococcus faecium* exhibited a more rapid rate of serum antigen specific IgG production and an increase in total IgA in the jejunum than those fed a control diet.

## 4 Conclusion

Wild type and mutant *Weissella confusa* produced EPS with different sugar moiety and composition. EPS were heteropolymeric in nature. The EPSWWC and EPSMWC had antioxidant capacity and there was variation in their activity. The antioxidant activity increases in a dose dependent manner. The EPS produced by wild type and mutant *Weissella confusa* had immunomodulatory potential against the treated mice. The EPSWC stimulated highest production of IgG and IgM while EPSWWC stimulated the highest production of IgA.

## Conflicts of interest

None.
